# Genome-wide analysis of fatty acid desaturase genes in rice (*Oryza sativa* L.)

**DOI:** 10.1038/s41598-019-55648-z

**Published:** 2019-12-19

**Authors:** Zhiguo E, Chen Chen, Jinyu Yang, Hanhua Tong, Tingting Li, Lei Wang, Hongqi Chen

**Affiliations:** 1 Key Laboratory of Rice Biology, China National Rice Research Institute, Hangzhou, 310006 China; 2 Key Laboratory of Plant Functional Genomics, Ministry of Education/Key Laboratory of Crop Genetics and Physiology of Jiangsu Province/College of Agriculture, Yangzhou University, Yangzhou, 225009 China

**Keywords:** Comparative genomics, Plant molecular biology

## Abstract

Fatty acid desaturases can catalyze saturated or unsaturated fatty acids to form a double bond at various locations in the hydrocarbon chain. In the present study, a total of 20 full-length desaturase genes were identified from rice genome. An exhaustive analysis was performed to describe their chromosomal locations, gene structures, phylogeny, *cis*-regulatory elements, sub-cellular localizations and expression patterns. The rice desaturase genes were distributed on ten of 12 chromosomes and phylogenetically classified into six subfamilies with the *Arabidopsis* counterparts, FAB2, FAD2, FAD3/7/8, FAD6, DES1 and SLD1. Among of them, 9 members were expanded via chromosomal tandem or segmental duplications. The gene structures and motif constituents were evolutionarily conserved in the same subfamilies. The majority of desaturase genes showed tissue-specific expression patterns and response to abiotic stresses and hormones based on microarray data and qRT-PCR analyses. This study will provide useful clues for functional validation of desaturase genes and contribute to produce nutritionally important fatty acids by genetic modification in rice.

## Introduction

Rice is one of the most important staple crops, provides almost half of the world’s population a dietary source of energy^1^. Apart from starches and proteins, rice grains contain a low proportion of lipids consisted mostly in the bran. Rice bran oil (RBO) extracted from rice bran is a commercial valuable by-product of milling, consisting of various fatty acids such as 36% oleic acid, 37% linoleic acid, 18% palmitic acid, 2.4% stearic acid, 1.5% linolenic acid and so on. RBO is as effective as other commonly used vegetable oils in lowering plasma cholesterol level^2^. The oxidative stability and nutritional value of RBO, which are already being considerable of commercial importance, are also affected by the levels of fatty acids^3^.

Fatty acid desaturases catalyze saturated or unsaturated fatty acids to form a double bond at various locations in the hydrocarbon chain^4^. They exist very widely in eukaryotic cells and contain three or two conserved histidine regions. Histidine-rich motifs are thought to form a part of the diiron center where oxygen activation and substrate oxidation happen^5–7^. The desaturation processes occur in both the endoplasmic reticulum (ER) membrane and the plastid membrane through two distinct pathways^7,8^. The genes encoding ER- and plastid-localized desaturases have been cloned and characterized from many plant species up to now. In rice, *OsSSI2* encoding a stearoyl acyl carrier protein fatty-acid desaturase, and is involved in producing oleic acid from stearic acid. Suppression of *OsSSI2* enhances resistance to blast and leaf blight diseases in rice^9^. A microsomal Δ12 fatty acid desaturase gene designated as *OsFAD2-1*, is responsible for the conversion of oleic acid into linoleic acid, and can improve the tolerance of rice to low temperature stress^10–12^. *OsFAD3* encode a ω-3 (Δ-15) fatty acid desaturase localized to the ER membrane, which catalyzed linoleic acid conversion to α-linolenic acid in rice seeds^13,14^. Chloroplast-localized OsFAD7 and OsFAD8 have also ω-3 desaturase activity for forming trienoic fatty acids and they negatively regulate the disease resistance against *Magnaporthe grisea*, in addition, OsFAD8 plays a significant role in stress tolerance at low temperature^14–16^.

Currently, the rice genome has been deeply sequenced and assembled, approximately 373 Mb captured in 12 chromosomes, and its 39102 predicted gene loci annotated by MSU-RGAP are publicly available^17^. So, rice has become the primary model for cereal species in plant science research, and its genome and annotated genes facilitate comparative genomic studies which will help to discover novel genes and explain some biological problems in silico^18^. In the present study, although several orthologous genes encoding fatty acid desaturases from rice were identified, our understanding of the biological function of the majority of these enzymes is limited. As such, there is an urgent need for an entire feature about the fatty acid desaturase family. This study provides a global overview of the desaturases which contains the gene structures, chromosome locations, phylogeny, and the expression profiling resulting from various organs/tissues of rice. The identification of novel desaturases will give us new insights into the pathways involved in unsaturated fatty acid metabolism and signaling transduction in rice. Moreover, the characterization of desaturases from rice will offer scientists abundant candidate genes for the production of nutritionally beneficial fatty acids in transgenic crops.

## Results and Discussion

### Identification of fatty acid desaturase genes in rice genome

We used several bioinformatics resources in our efforts to thoroughly explore the entire *FAD* gene family in rice. 29 gene models were obtained through FAD domain searching (FA_desaturase, PF00487; FA_desaturase_2, PF03405) with the MSU Rice Genome Annotation Project Database (MSU-RGAP, http://rice.plantbiology.msu.edu/analyses_search_domain.shtml). In the Rice Annotation Project Database (RAP-DB, http://rapdb.dna.affrc.go.jp/), using a keyword search for “fatty acid desaturase”, 10 genes were identified. The orthologous protein sequences of *Arabidopsis* desaturases were used as queries in BLASTP searches against the rice genome entries in the RAP-DB databases. Following removal of the redundant sequences and eliminating alternate splice variants of the same gene, a total of 20 fatty acid desaturase genes were thus identified in rice. Among of them, nine members homologous with *Arabidopsis FAB2.1* were designated as *OsFAB2-1~9*. Analogously, four FAD2 subfamily members, four FAD3/FAD7/FAD8 subfamily members, and one each in DES1, SLD1 and FAD6 subfamily were named. The detailed information about each gene locus, FL-cDNA, ORF length, and characteristics of corresponding proteins are detailed in Table 1.

**Table 1:** The general information and sequence characterization of 20 fatty acid desaturase genes in rice.

Gene Name/Alias	Accession Number		Intros	Protein ^a^			subcellular localization^b^	*Arabidopsis* ortholog locus
	RGAP Locus	FL-cDNA		Size(aa)	MW(D)	pI		
*OsFAD2-1*	LOC_Os02g48560	AK061506	1	389	44350.1	8.23	ER	*FAD2*(AT3G12120)
*OsFAD2-2*	LOC_Os07g23430	AK105371	1	470	52845.2	9.35	plasma membrane	*FAD2*(AT3G12120)
*OsFAD2-3*	LOC_Os07g23410	AK070559	1	391	44981.0	9.51	ER	*FAD2*(AT3G12120)
*OsFAD2-4*	LOC_Os07g23390	NA	1	224	24677.1	12.61	plasma membrane	*FAD2*(AT3G12120)
*OsFAD3-1*	LOC_Os11g01340	AK242740	7	385	43902.4	7.48	ER	*FAD3*(AT2G29980)
*OsFAD3-2*	LOC_Os12g01370	AK071185	7	386	43899.5	8.01	ER	*FAD3*(AT2G29980)
*OsFAD6*	LOC_Os08g34220	AK060449	9	455	52243.7	9.51	chloroplast	*FAD6*(AT4G30950)
*OsFAD7*	LOC_Os03g18070	AK058242	7	459	50772.1	7.66	chloroplast	*FAD7*(AT3G11170)
*OsFAD8*	LOC_Os07g49310	AK061531	6	414	47012.0	8.73	chloroplast	*FAD8*(AT5G05580)
*OsFAB2-1*	LOC_Os01g65830	AK059526	1	382	42942.9	7.10	chloroplast	*FAB2.1*(AT1G43800)
*OsFAB2-2*/*OsSSI2*	LOC_Os01g69080	AK058979	2	401	45320.7	7.07	chloroplast	*FAB2.2*(AT2G43710)
*OsFAB2-3*	LOC_Os02g30200	AK069683	3	388	42513.3	7.02	chloroplast	*FAB2.2*(AT2G43710)
*OsFAB2-4*	LOC_Os03g30950	AK070282	1	419	45365.5	7.94	chloroplast	*FAB2.4*(AT3G02620)
*OsFAB2-5*	LOC_Os04g31070	AK065340	2	391	44341.4	7.01	chloroplast	*FAB2.2*(AT2G43710)
*OsFAB2-6*	LOC_Os03g53010	NA	0	264	30087.6	10.47	mitochondrion	*FAB2.2*(AT2G43710)
*OsFAB2-7*	LOC_Os06g30780	NA	0	190	22534.2	12.46	mitochondrion	*FAB2.4*(AT3G02620)
*OsFAB2-8*	LOC_Os08g09950	AK105852	1	423	46491.2	7.98	chloroplast	*FAB2.3*(AT3G02610)
*OsFAB2-9*	LOC_Os08g10010	AK241294	1	405	44829.0	7.41	mitochondrion	*FAB2.3*(AT3G02610)
*OsDES1*	LOC_Os02g42660	AK101968	1	329	37868.6	8.93	plasma membrane	*DES1*(AT4G04930)
*OsSLD1*	LOC_Os09g16920	AK058543	0	467	51723.8	8.55	plasma membrane	*SLD1.1*(AT2G46210)

^a^Protein characterization of fatty acid desaturases obtained from RGAP.

^b^Subcellular location prediction using CELLO2GO Server (http://cello.life.nctu.edu.tw/cello2go/).

aa, amino acids; MW, molecular weight; pI, isoelectric point; NA, not available.

**Figure 1:**
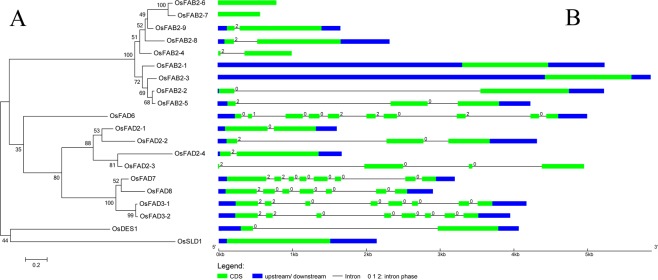
Phylogenetic relationship and intron-exon structures of desaturase genes. (**A**), Phylogenetic tree of 20 desaturase genes using maximum-likelihood methods. The MEGA software (version 7.0.25) was used to generate the phylogenetic tree. Scale bar represents 0.2 amino acid substitution per site. The proteins on the tree can be divided into six distinct subfamilies. The number in the line represents the goodness of fit (%). (**B**), intron-exon structures of desaturase genes. The numbers 0, 1 and 2 indicate the phase of introns.

The numbers and positions of exons and introns of each desaturase gene were determined by the comparison of the CDS sequences and the corresponding genomic sequences via using the Gene Structure Display Server website (GSDS, http://gsds.cbi.pku.edu.cn/)^19^. Three gene (*OsFAB2-*6, *OsFAB2-7* and *OsSLD1*) lacked introns; the number of introns in the coding sequences of the other 17 genes ranged from one to nine (Fig. 1B). Four genes (*OsFAB2-3*, *OsFAB2-6*, *OsFAB2-7* and *OsFAD2-4*) had no untranslated regions. Most orthologous genes tended to share similar exon-intron structure and transcript length. For example, *OsFAD3-1* and *OsFAD3-2* each contain seven introns and eight exons, and are all nearly 1155 bp in length (Fig. 1B).

### Chromosomal localization and gene duplication

**Figure 2:**
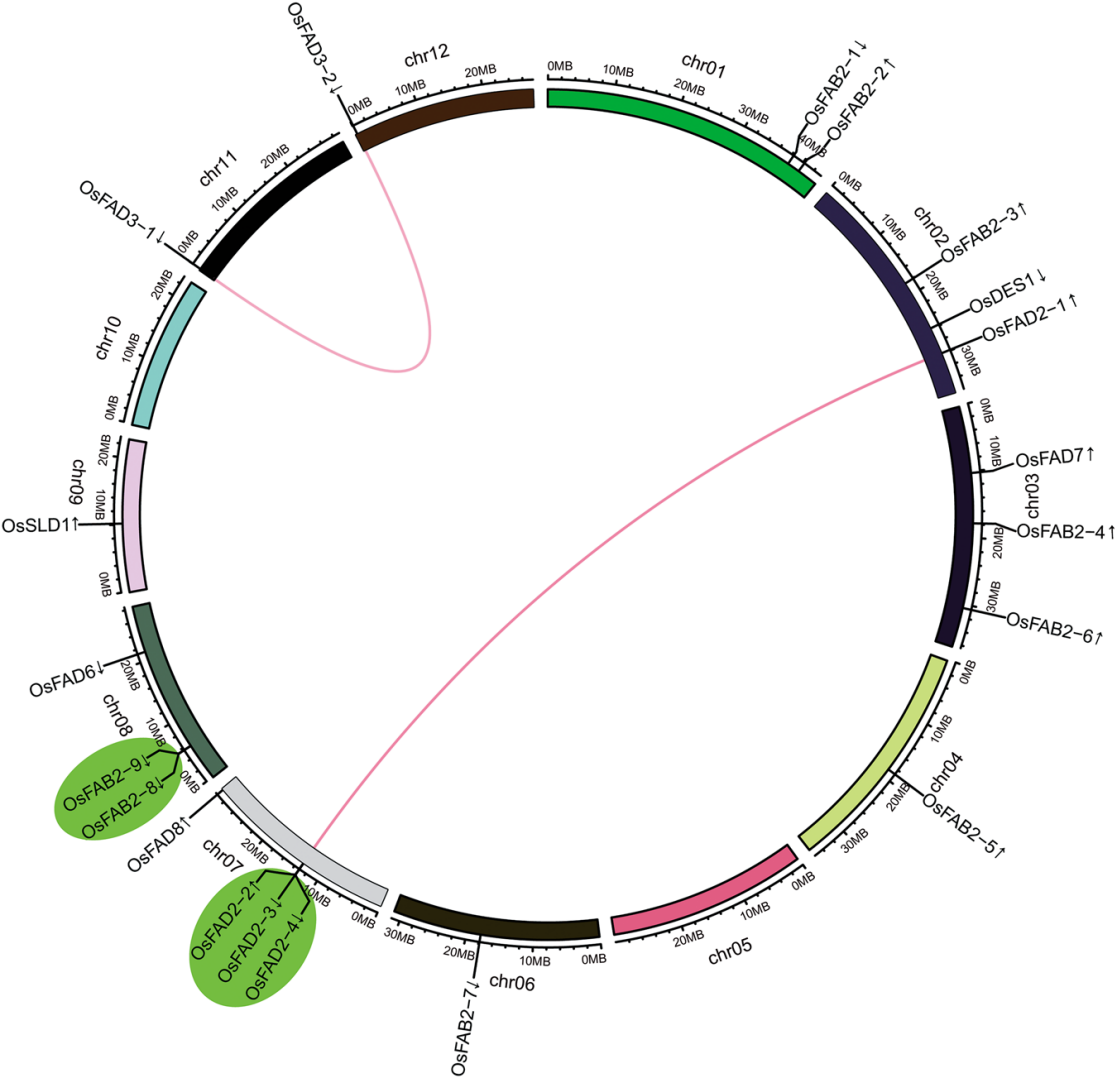
Chromosomal localization and gene duplication events of desaturase genes. The circlize package in R (version 3.6.1) was used to generate the circos map. Respective chromosome numbers are indicated at the center of each arc. The scale on the arc is in megabases (Mb). The segmental duplication genes are connected by straight line in color. The tandemly duplicated genes are shaded with ovals in light blue. The arrows next to gene names show the direction of transcription.

Based on the MSU-RGAP loci coordinates (http://rice.plantbiology.msu.edu/cgi-bin/gbrowse/rice/), the accurate locations and orientation of the fatty acid desaturase genes on the rice chromosomes were determined. All of the 20 genes were distributed on ten of 12 chromosomes, excluding chromosomes 5 and 10. Rice chromosome 7 has four desaturase genes, chromosomes 2, 3 and 8 each have three, chromosome 1 has two and chromosomes 4, 6, 9, 11 and 12 each only contain one member (Fig. 2).

To explore the impacts of duplication events during evolution on the expansion of the fatty acid desaturase family, we investigated tandem and segmental duplications. According to the criterion of separation by less than 5 intervening genes and >50% homology at protein level, a total of 5 genes fell into two groups were tandemly duplicated. Besides, two pairs of genes were found to be segmentally duplicated. So, among the 20 desaturase genes, 45.0% (9 of 20) were involved in duplication events, including 5 genes with tandem duplication and 4 genes with segmental duplication. The tandemly duplicated genes were on chromosomes 7 and 8 (Fig. 2). Interestingly, four genes (*OsFAD2-1/-2/-3/-4*) were expanded through both tandem and segmental duplications. These results showed that the gene duplication events contributed to the expansion of the desaturase family in rice.

### Multiple sequence alignment and phylogenetic analysis

To clearly apprehend the sequence characteristics of fatty acid desaturases in rice, we performed a multiple sequence alignment using the deduced amino acid sequences of the 20 proteins. The 20 gene products contained typical histidine-rich boxes (Table 2), which accorded with the standard of different types of desaturase genes. For instance, majority of genes from FAD3/7/8 subfamily matched the standard for plastidial Δ12 desaturase, namely, GHDCXH, HXXHH and HVXHH; two histidine-boxes of nine FAB2 subfamily members were consistent with these of stearoyl-[acyl-carrier-protein] desaturases characterized by EENRHG and DEXRHE. To be sure, an exception was that LOC_Os07g23390 appeared to be truncated at the C-terminus missing the third histidine-rich box, so no FAD domain (either PF00487 or PF03405) could be detected in Pfam database. But in keeping with previous published article^11^, LOC_Os07g23390 was still considered as OsFAD2*-*4.

**Table 2:** Conserved histidine-rich boxes of fatty acid desaturases in rice.

Subfamily	H-box 1	H-box 2	H-box 3
FAB2	WT(S)AE(K)ENR(H)HGD	AA(S)DEKRHE	/
FAD2	WV(I)IA(G)HECGHHAFS	FSWKYS(T)HR(Q)RHHSNT	TDTHVXHHLFP(S)
FAD3/7/8	WALFVLGHDCGHGSFS	YHGWRISHRTHHQNHGH	HHDIGTHVIHHLFPQIPHYHL
FAD6	FFVIGHDCAHRKSFS	EPWRFKHDRHHAKTN	HDINVHVPHHISPR
SLD1	GHDSGHH	WWKCNHNTHHIACNSLD	GGLQFQIEHHLFPRLPR
DES1	NLFLAIHELSHNLAF	FQKYHLEHHRFQGVDGID	HVGYHNEHHDFPRIPG

To examine in detail the phylogenetic relationship and functional divergence among the 20 desaturases in rice, a phylogenetic tree was constructed (Fig. 1A). The 20 desaturases were classified into 6 distinct subfamilies; the subfamilies were named according to their identity to *Arabidopsis* desaturases and included subfamilies FAB2, FAD2, FAD3/7/8, FAD6, DES1 and SLD1. The rice homologs of Arabidopsis AtFAD5 and AtASD1/2 were not present. Among the 6 subfamilies, subfamily FAB2 with nine members was the largest (Fig. 1A).

In order to identify orthologous genes between *Arabidopsis* and rice, an integrated phylogenetic tree composed of members of the two species was constructed (Fig. S1). In this analysis, similar subfamilies were formed as compared with the evolutionary tree of rice. Except for the subfamily FAD5/ADS1 absent in rice genome, each clade of a distinct subfamily consisted of desaturases from both rice and *Arabidopsis*. Furthermore, the orthologues between *Arabidopsis* and rice in subfamily FAB2, FAD2, FAD3/7/8, FAD6, DES1, and SLD1 showed very high sequence identities. These results indicated that the formation of desaturase family in *Arabidopsis* and rice occurred before the split of dicots and monocots.

### Expression analysis of desaturase genes during development

**Figure 3:**
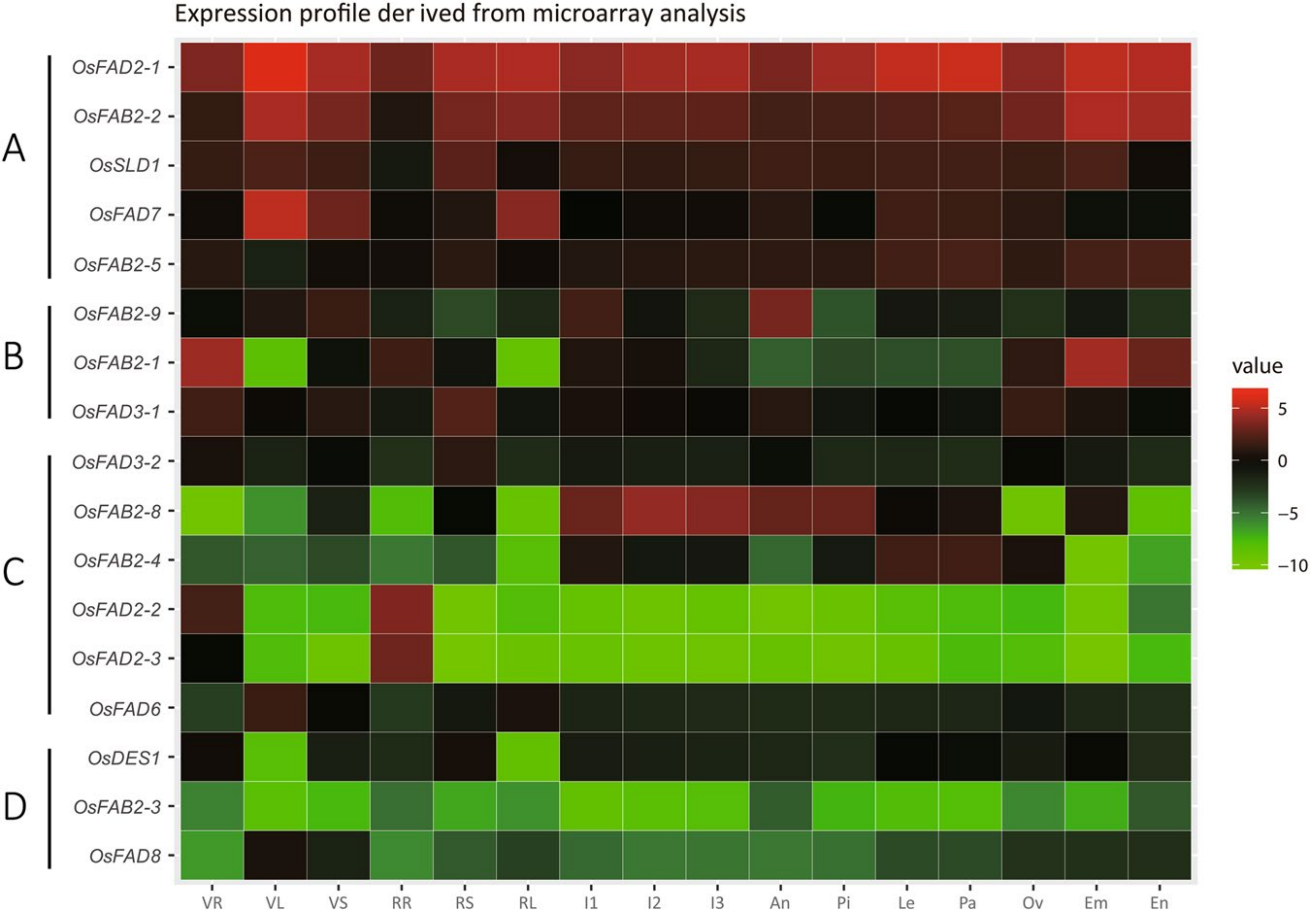
Expression profiles of desaturase genes in various tissues/organs. The ggplot2 package in R (version 3.6.1) was used to generate the heat map. The microarray data sets (RXP_0001) of fatty acid desaturase genes expression in tissues at various developmental stages were analyzed. Color key represents average log2 expression values of desaturase genes. The color scale (representing average log signal values) is shown at the right. Samples are mentioned at the top of each lane: VR, roots at vegetative stage (27 days after transplanting, same as below); RR, roots at reproductive stage (76 days after transplanting, same as below); VL, vegetative leaf blades; RL, reproductive leaf blades; VS, vegetative leaf sheaths; RS, reproductive stems; different stages of inflorescences development: I1, 0.6–1.0 mm; I2, 3.0–4.0 mm; I3, 5.0–10.0 mm; An, 0.3–0.6 mm anthers; Pi, pistils from 5–10 cm panicles; Le, lemmas from 1.5–2.0 mm florets; Pa, paleae from 1.5–2.0 mm florets; Ov, ovaries at 1 day after flowering; Em, embryos at 7 days after flowering; En, endosperms at 7 days after flowering. Genes that share similar expression patterns are divided into four groups: (**A**) high expression in all examined organs; (**B**) relatively high expression levels in particular tissues; (**C**) abundant expression in vegetative or reproductive organs; (**D**) low expression in all examined tissues.

To gain valuable insights into the possible function of desaturase genes during rice development, we analyzed their expression patterns in various organs/tissues at different developmental stages using microarray data (http://ricexpro.dna.affrc.go.jp/GGEP/). Data for the following tissues was examined: vegetative roots (VR), reproductive roots (RR), vegetative leaf blades (VL), reproductive leaf blades (RL), vegetative leaf sheaths (VS), reproductive stems (RS), inflorescences (I1-I3), anthers (An), pistils (Pi), lemmas (Le), paleae (Pa), ovaries (Ov), embryos (Em), and endosperms (En). A heat map generated from the average log signal values for the 17 desaturase genes in selected tissues summarizes the differential expression patterns of these genes (Fig. 3). The expression patterns of these 17 desaturase genes can be divided into four major groups. Group I includes 5 genes that show high expression levels in all examined tissues, expect for two genes with relatively low expression in certain developmental stages (*OsFAB2-5* in VL, *OsSLD1* in RR). Group II consists of 3 genes which show relatively high expression levels in particular vegetative and reproductive tissues: *OsFAB2-9* in VS, I1, and anthers; *OsFAB2-1* in VR, RR, ovaries, embryos and endosperm; *OsFAD3-1* in VR, VS, RS and ovaries. Group III comprises 6 genes that show abundant expression level in vegetative or reproductive organs: *OsFAD2-2* and *OsFAD2-3* in roots; *OsFAD3-2* in stems; *OsFAB2-*8 in VL; OsFAB2-8 in I1-3, anthers and pistils; *OsFAB2-4* in I1, lemmas and paleae. The last remaining genes, *OsFAB2-3*, *OsFAD8* and *OsDES1*, belong to Group IV, shows relatively low expression level in all investigated tissues.

**Figure 4:**
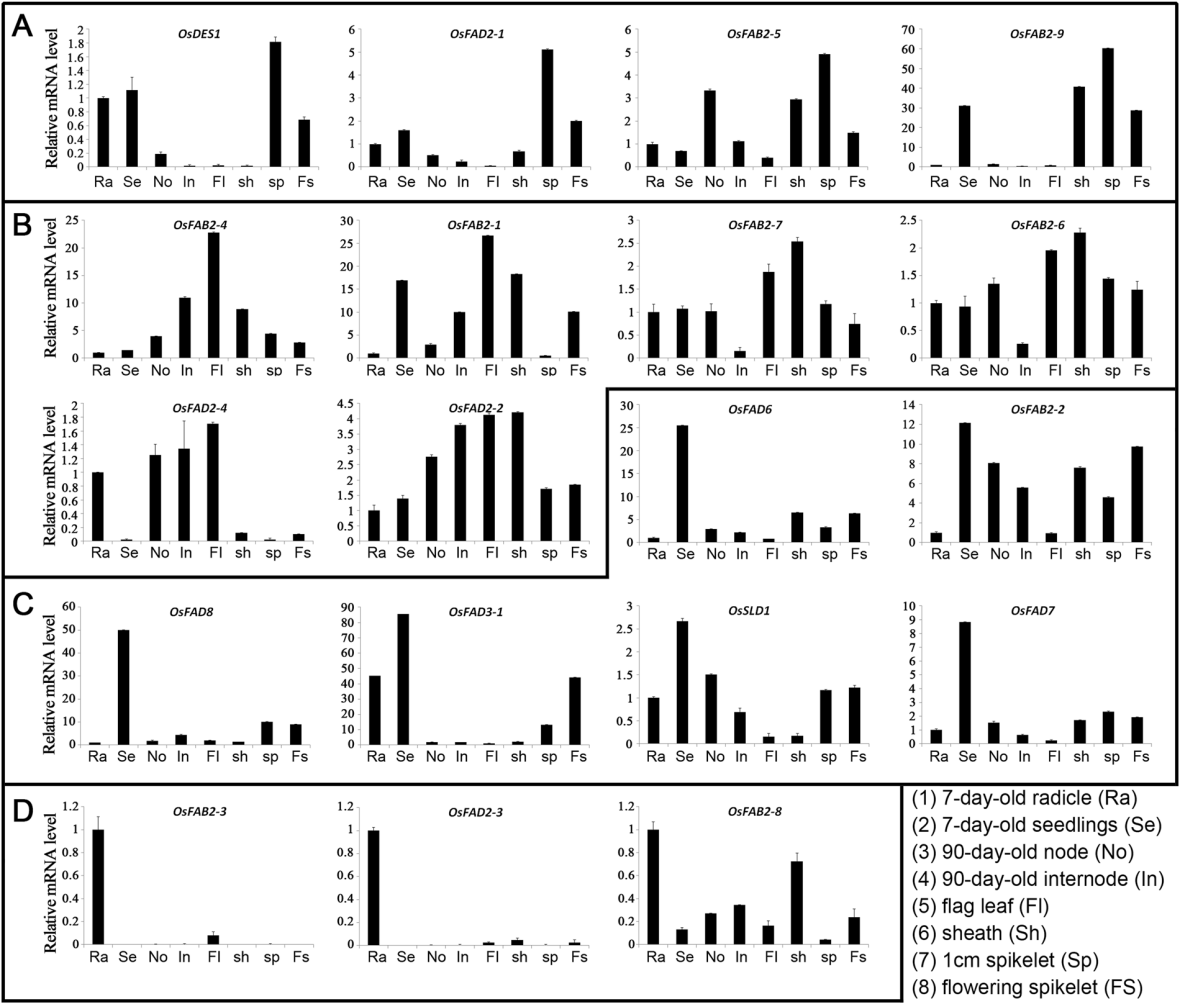
Real-time PCR analysis of tissue-specific expression of the desaturase genes. The ggplot2 package in R (version 3.6.1) was used to generate the cluster bar chart. Relative mRNA levels of individual genes normalized to *Os03g0234200* are shown. The genes with preferential expression levels in spikelets (**A**), flag leaves and sheaths (**B**), seedlings (**C**) and radicles (**D**) were showed. Error bars indicate standard deviations of independent biological replicates (n = 3 or more).

To verify the results of above digital expression analyses, we employed real-time PCR analysis to confirm the expression levels of the desaturase genes in rice. It was demonstrated that the real-time PCR results were generally consistent with the microarray data. For example, *OsFAB2-2/5* and *OsSLD1* are constitutively expressed; *OsFAD6* and *OsFAB2-3* are predominantly expressed in seedlings and roots, respectively (Fig. 4). Nevertheless, there are still some exceptions. For example, *OsFAD8* and *OsFAD2-2* are relatively high expressed in all investigated tissues, especially, *OsFAD8* in seedlings (Fig. 4).

In *Arabidopsis*, expression patterns of fatty acid desaturase gene family were also reported. Chi *et al*. 2011 reported organ-specific and developmental expression profiles of 16 desaturase genes in *Arabidopsis*, 12 of 16 genes were expressed in specific tissues^7^. Interestingly, serval pairs of homologous genes in *Arabidopsis* and rice shared similar expression profiles in development process. For example, *FAD2* was relatively highly expressed in all investigated tissues, and *DES1* and *FAB2.3* were lowly expressed. Of course, some homologous genes showed inconsistent expression patterns, for example, *SLD* showed high expression levels in all tissues expect for reproductive roots in rice, but was barely expressed in *Arabidopsis*. The similarities and differences of expression profiles of these orthologous genes may imply their functional conservatism and evolution.

### Regulation of desaturase gene expression in response to abiotic stresses

**Figure 5:**
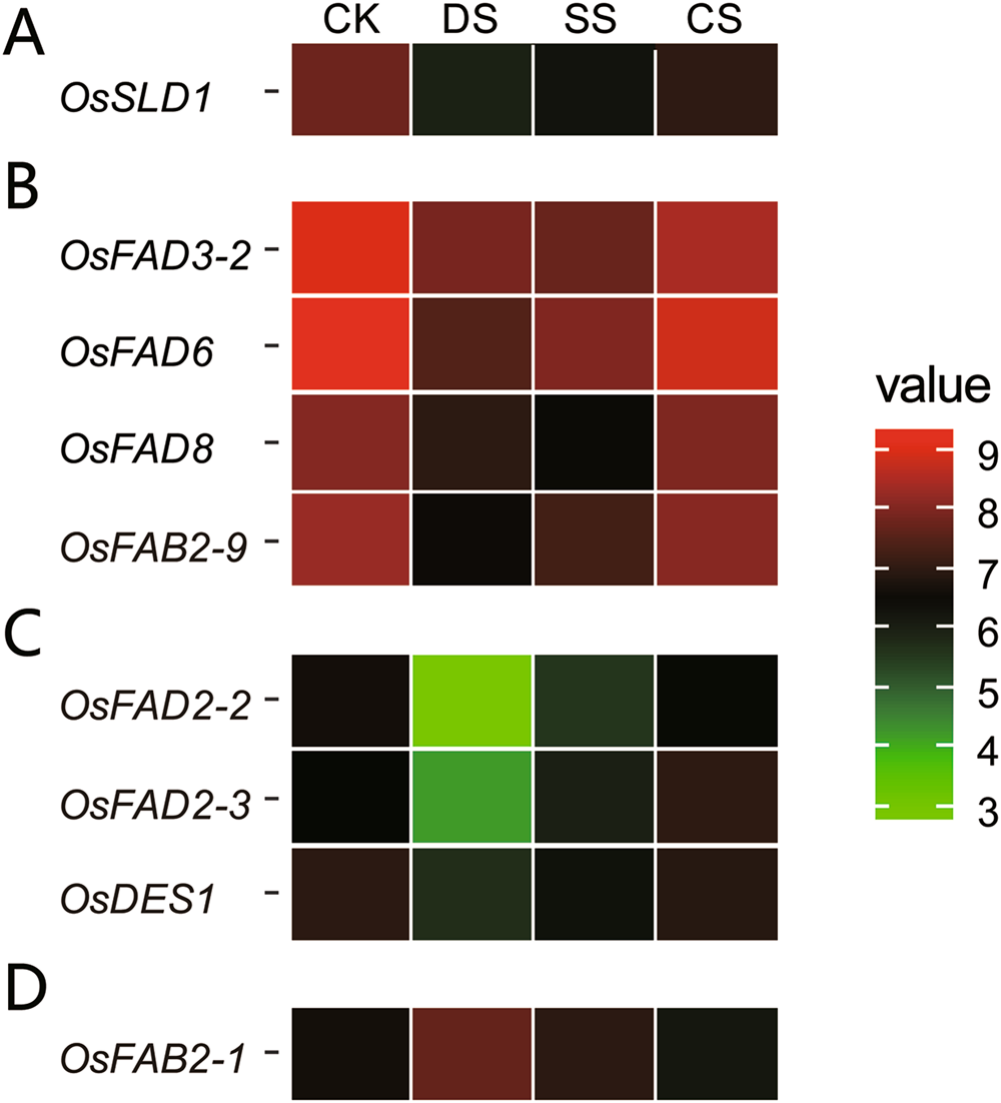
Differential expression profiles of desaturase genes under abiotic stresses. The ggplot2 package in R (version 3.6.1) was used to generate the heat map. The microarray data set (GSE6901) of gene expression in response to various abiotic stresses was used for cluster display. The average log2 fold change of desaturase gene expressions under control and various stress conditions (indicated at the top of each lane) are exhibited by a heat map. (**A**) Down-regulated by all three stresses; (**B**) Down-regulated by both drought and salt stresses; (**C**) Down-regulated by drought stress; (**D**) Up-regulated by drought stress.

To examine the response of desaturase genes to various abiotic stresses, the microarray data (GSE6901) for 7-day-old seedlings treated with drought, salt and cold stress was analyzed. A total of 8 genes were significantly (P < 0.05) down-regulated and only one was up-regulated in at least one of the stress conditions (Fig. 5A–D). The transcriptional level of *OsSLD1* was down-regulated under all three stresses (Fig. 5A). Four genes (*OsFAD3-2*, *6*, *8* and *OsFAB2-*9) were significantly down-regulated by both drought and salt stresses (Fig. 5B). Three genes (*OsFAD2-2*, *OsFAD2-3* and *OsDES1*) were specifically down-regulated by drought stress (Fig. 5C). *OsFAB2-1* was up-regulated under drought stress (Fig. 5D). All in all, it appeared that cold stress had only very limited influence, but drought stress had a much higher effect on the expression of these desaturase genes. These results suggest that desaturase genes may be involved in drought signaling pathways and play important roles in responses to drought stresses by changing the content ratio of the various fatty acid compositions in rice.

### Differential expression of desaturase genes in response to hormones

**Figure 6:**
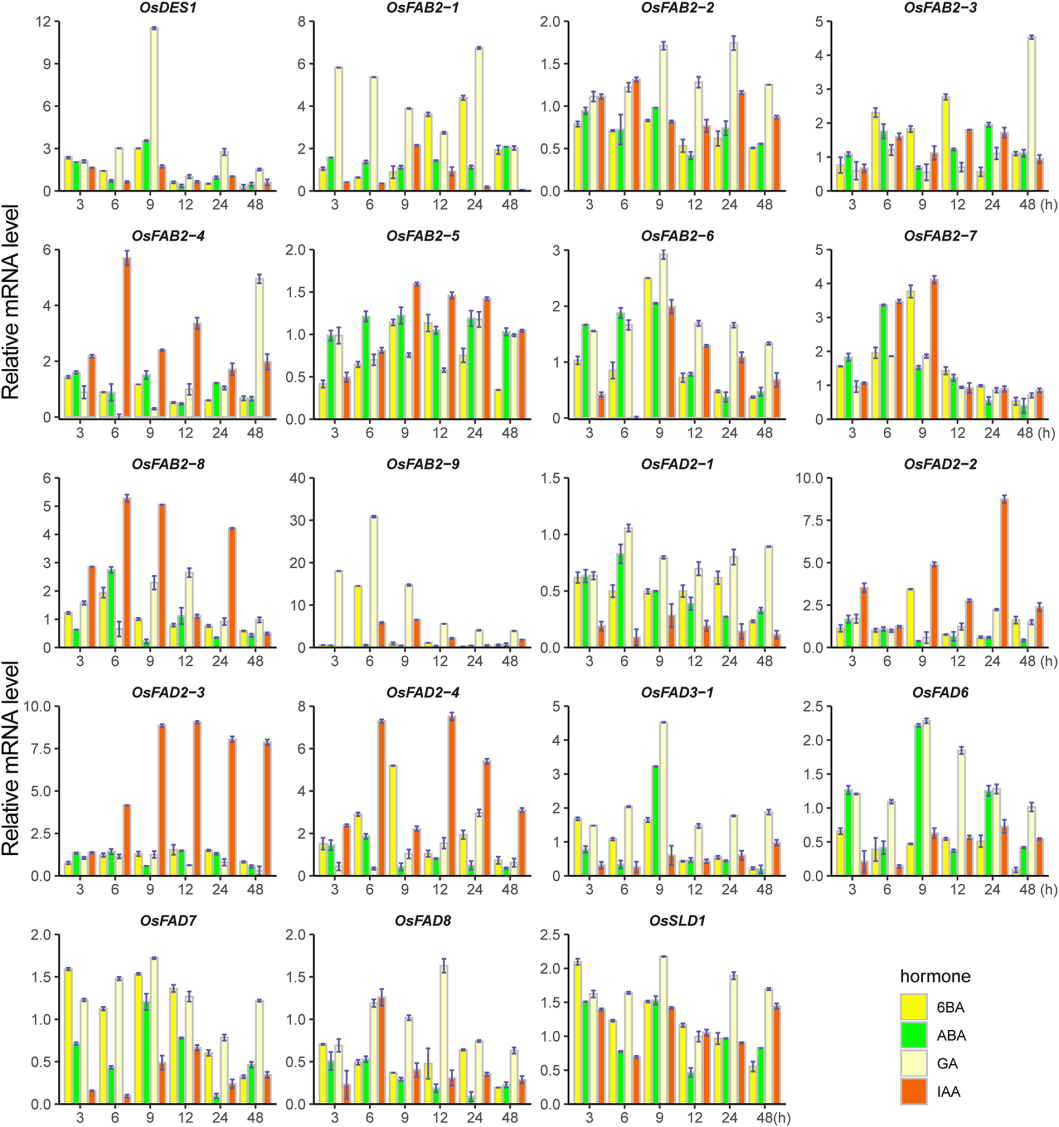
Expression analysis of desaturase genes under different hormone treatments. The ggplot2 package in R (version 3.6.1) was used to generate the cluster bar chart. X-axis indicates time course/treatment and Y-axes are scales of relative expression level. The expression levels of the control at all time points have been normalized to 1. Error bars indicate standard deviations of independent biological replicates (n = 3 or more). The ggplot2 package in R (version 3.6.1) was used to generate the bar chart map. IAA, indole-3-acetic acid; 6-BA, 6-Benzylaminopurine; GA, gibberellin acid; ABA, abscisic acid; h, hour.

To determine if desaturase genes in rice are involved in hormone signaling pathways, we investigated their expression profiles under different phytohormone treatments. Total RNA was isolated from seedlings of *Nipponbare* rice at three-leaf stage treated with ABA (Abscisic Acid), 6-BA (6-Benzylaminopurine), IAA (Indole-3-Acetic Acid) and GA (Gibberellin Acid), and the expression levels of the desaturase genes were evaluated using quantitative RT-PCR (Fig. 6). The results showed that the expressions of 8 genes (*OsSLD1*, *OsDES1*, *OsFAB2-1*, *OsFAB2-4*, *OsFAB2-8*, *OsFAB2-9*, *OsFAD2-2*, *OsFAD2-4*) were markedly down- or up-regulated (<50% or >2-fold) under at least one of the hormone treatments at 3 h time point, as compared with the untreated control. It implied that these genes may be involved in plant’s early response to the hormones.

Under IAA treatment, the transcript levels of four genes (*OsFAD2-1*, *OsFAD3-1*, *OsFAD6* and *OsFAD7*) and two genes (*OsFAB2-4*, *OsFAD2-4*) were decreased and increased at 6 sampling time points, respectively. Two genes (*OsSLD1*, *OsFAB2-5*) were almost unaffected by IAA. These data indicated that the various desaturase genes showed different induction kinetics in response to auxin.

Under GA treatment, four genes (*OsFAD3-1*, *OsFAD6*, *OsDES1*, *OsSLD1* and *OsFAB2-6*) were induced slightly at the early time points and reached peak value at 9 h, but were reduced at the later time points. The expressions of two genes (*OsFAB2-3*, *OsFAB2-4*) were repressed during the whole early-middle period, but were induced strongly at 48 h time point. Two genes (*OsFAB2-1*, *OsFAB2-9*) showed high express level at the whole time points. The results revealed that most of the desaturase genes were responsive to gibberellin.

Under ABA treatment, the expression levels of 10 genes (*OsFAB2-2*, *OsFAB2-6*, *OsFAB2-7*, *OsFAB2-8*, *OsFAD2-1*, *OsFAD2-4*, *OsFAD3-1*, *OsFAD6*, *OsFAD8* and *OsDES1*) showed different changes at the different time points of the treatment; that is to say, these genes were repressed (<50%) at some time points, but induced (>2-fold) at other time points. It illustrated that desaturase genes could play a complex role in abscisic acid signaling pathways in rice. Meanwhile, the expression patterns of quite a part of genes under 6-BA treatment were similar with the result of ABA treatment; for example, the transcript levels of eight genes (*OsSLD1*, *OsDES1*, *OsFAB2-1*, *OsFAB2-3*, *OsFAB2-6*, *OsFAB2-7*, *OsFAB2-9* and *OsFAD2-2*) were noticeable increased at particular time points, but decrease at other times. Moreover, the three genes (*OsFAB2-2*, *OsFAD2-1* and *OsFAD8*) were reduced throughout the treatment time courses; this is also consistent with ABA treatment.

Comparison of the induction kinetics of desaturase genes under different hormones stimulus revealed that the expression levels of the majority of genes increased under at least two hormones treatments. Three genes (*OsFAB2-6*, *OsFAB2-7* and *OsFAB2-8*) and one gene (*OsFAD2-1*) were up-regulated and down-regulated by all the four hormone treatments, respectively. The hormone-responsive expression profiles of this family suggested that almost all of the genes were responsive to the four hormones tested in these experiments expect *OsFAB2-5*, which was not responsive to ABA.

### Cis-regulatory elements in the promoter of desaturase genes

**Figure 7:**
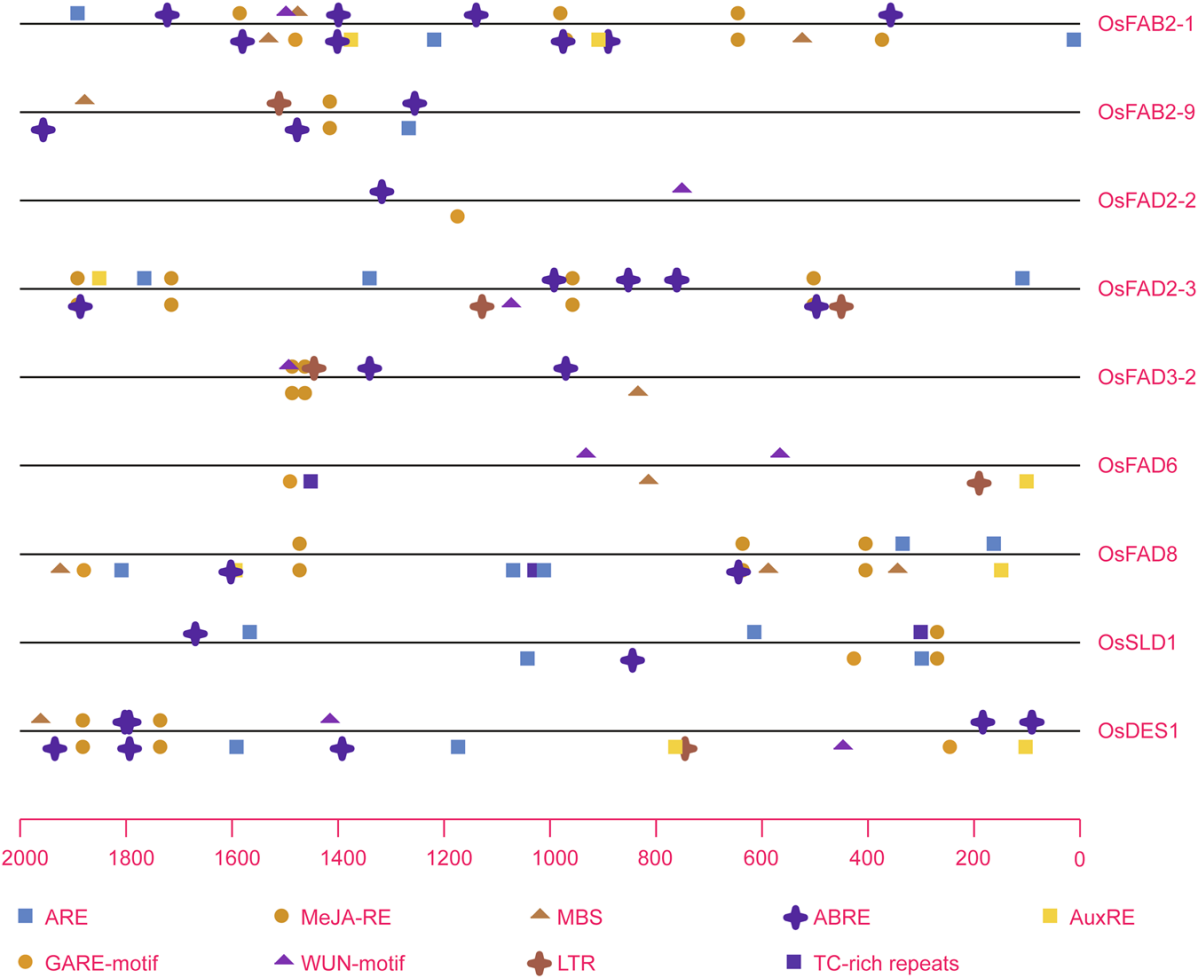
Promoter analysis of nine stress or hormone-responsive desaturase genes. Abiotic stress or hormone-related *cis*-regulatory elements of the -2 Kb 5′ upstream regions of desaturase genes are shown. *Cis*-elements in the template strand are indicated above the line, and those in the complementary strand are below the line.

Conserved regulatory elements in promoter sequences were involved in response to various growth factors and environmental stresses. Here, the promoter sequences (~2.0 kb) of nine abiotic stress and hormone-induced desaturase genes (*OsFAB2-1*/*9*, *OsFAD2-2/3*, *OsFAD3-2*, *OsFAD6*, *OsFAD8*, *OsSLD1* and *OsDES1*) were selected to compare with each other (Fig. 7). It was observed that dozens of different cis-acting elements predicted by PlantCARE were discovered. Among them, several *cis*-acting elements, like G-boxes, CAAT-boxes and TATA-boxes and so on, were mutual in nine examined genes. To more intuitively explore the promoter regions, a total of nine developmental or stress-related *cis*-regulatory elements were used for promoter analysis, including abscisic acid responsive element (ABRE), anaerobic response element (ARE), auxin-responsive element (AuxRE), gibberellin-responsive element (GARE-motif), low temperature responsive element (LTR), myb-binding site involved in drought-inducibility (MBS), Methyl jasmonate-responsive element (MeJA-RE), defense and stress responsive element (TC-rich repeat) and wounding and pathogen responsive element (WUN-motif). The results showed that eight out of nine examined genes except *OsFAD2-2* possessed multiple ABRE elements and at least four different other regulatory elements in their promoter regions; it was in accordance with their expression profiles in response to abiotic stress and ABA treatment. In addition, each gene contained a number of various light responsive elements, such as Box 4, G-Box, GT1-motif, TCT-motif, Sp1, and so forth. It was estimated that desaturases might be also involved in light response.

Further, the PlantPAN 3.0 database was used to screen the predicted transcription factors (TF) and their binding sites of the nine desaturase genes. Subsequently, the TFs from PlantCARE and *cis*-acting elements from PlantPAN were integrated into a table, depending on the promoter motifs and their positions (Table S1). A total of 189 members of thirteen TF families were identified. Among them, numerous bZIP, bHLH, Homeodomain and SBP transcription factors were predicted to bind the ABRE and MeJA-RE elements. Similarly, the WUN-motif elements could be bound by NF-YB, NAC and AT-Hook transcription factors. Besides, AP2, MYB and WRKY etc. transcription factors were able to interact with other regulatory elements. The results indicated that these *cis*-acting elements and corresponding *trans*-acting factors may be activated in response to hormones and environmental stresses.

## Materials and Methods

### Identification of fatty acid desaturase genes in rice

To explore all of the putative desaturase members in rice, three approaches were employed, as described in detail previously^20^. First, the five protein sequences of *Arabidopsis* desaturases (FAD2/AT3G12120, FAB2/AT2G43710, DES1/AT4G04930, SLD1/AT2G46210 and ADS1/AT1G06080) were used as queries to execute BLASTP grogram against rice protein database (https://rapdb.dna.affrc.go.jp/tools/blast) with default parameters except for E-value set to e^−10^ (default value is 0.1). Secondly, two Pfam domains (PF00487 and PF03405) searching was performed using the MSU-RGAP website (http://rice.plantbiology.msu.edu/analyses_search_domain.shtml). Thirdly, In the Rice Annotation Project Database (RAP-DB, http://rapdb.dna.affrc.go.jp/), using a keyword search for “fatty acid desaturase”. After removing the redundant sequences, the remaining protein sequences were defined as putative fatty acid desaturases. The genomic, transcript, coding sequence (CDS) and peptide sequences of identified genes were retrieved from MSU-RGAP (http://rice.plantbiology.msu.edu/), and full-length cDNA accessions were obtained from NCBI (https://www.ncbi.nlm.nih.gov/). The gene structures of the desaturase members were analyzed using the GSDS2.0 website (Gene Structure Display Server, http://gsds.cbi.pku.edu.cn/)^19^.

### Sequence analysis of desaturase genes/proteins

The physical positions of desaturase genes given by the MSU-RGAP database were used to chromosomal mapping, and their distribution on chromosomes was drawn by R package circlize^21^. Multiple sequence alignment and conservation analysis were performed using CLC Genomics Workbench 12.0 with default parameters. The un-rooted phylogenetic tree of all desaturases was constructed by MEGA 7.0 using the neighbor-joining (NJ) method with 1000 bootstrap replicates, poisson model and complete deletion^22^. Main criteria used for analyzing potential segmental gene duplication events included: (a) length of alignable region covers > 75% of longer gene, and (b) similarity of aligned regions > 75%^23^. Subcellular localization was predicted by CELLO2GO (http://cello.life.nctu.edu.tw/cello2go/)^24^.

### Promoter analysis

The 2000 base pairs upstream from the ATG translational start codon of the desaturase genes were extracted from the RAP-DB database. The upstream sequences were subsequently scanned in the PlantCARE website (http://bioinformatics.psb.ugent.be/webtools/plantcare/html/) and PlantPAN 3.0 database (http://plantpan.itps.ncku.edu.tw/promoter.php) to analyze the presence of various *cis*-acting regulatory elements and corresponding *trans*-acting factors^25,26^.

### Plant material and treatments with hormones

As described in detail previously^20^, in order to evaluate the spatio-temporal expression profiles of desaturase genes, the rice seedlings of the cultivar Nipponbare were grown in the field during the normal growing season at 30–34: 22–26 °C (day: night) and 80–95% humidity with a photoperiod of 14 h. The eight materials tested in the expression analysis were: (1) 7-day-old radicle (R); (2) 7-day-old seedlings (Se); (3) 90-day-old node (N); (4) 90-day-old internode (In); (5) flag leaf (Fl); (6) sheath (Sh); (7) 1 cm spikelet (Sp); and (8) flowering spikelet (Fp). For hormones treatments, rice seeds were immersed in water at 37 °C for 30 h, and then were sown on a plastic net that was floating on a nutrient solution in a growth chamber at 28 °C [light: dark = 14 h: 10 h]. Then, seedlings at three-leaf stage were transferred into containers and treated with 10 μM IAA, 10 μM 6′-BA, 10 μM GA, 25 μM ABA or non-treatment for control, and placed in a 28 °C illumination incubator^20^. At 3, 6, 9, 12, 24, 48 h after these treatments, seedlings were harvested.

All materials harvested were immediately frozen in liquid nitrogen and stored at −80 °C prior to RNA extraction.

### Digital expression and qRT-PCR analysis of desaturase genes

The microarrays expression data of desaturase genes were extracted from the Rice Expression Profile Database (http://ricexpro.dna.affrc.go.jp/), and were used to analyze expression profiles in various organs at different developmental stages (RXP_0001). Additionally, the total RNA isolation from various tissues, cDNA synthesis and quantitative RT-PCR were performed to analyze the expression profiles under hormones treatments. Real-time PCR was performed using a SYBR Green Realtime PCR Master Mix (TOYOBO) on an ABI StepOnePlus Real-Time PCR System. The 2^−△△CT^ method was used to analyze relative changes in gene expression^27^. The rice ubiquitin gene (Os03g0234200) was used as a reference in the experiment. The gene-specific primers were listed in Table S2.

## Conclusions

Making an intensive study of fatty acid desaturase genes will be conducive to deep understanding of the gene family. The present study investigated thoroughly the fatty acid desaturase gene family in rice. We identified several tissue-specific, abiotic stress and hormone-responsive desaturase genes and analyzed their chromosomal locations, gene structures and phylogeny. Tandem and segmental duplications of desaturase genes and the presence of various *cis*-regulatory elements in the promoter were also analyzed. The evidence of altered expression of desaturase genes in response to hormone and stress may explain their role for developmental processes and drought, salt or cold stress tolerance. However, their true functions in growth, development and stress tolerance require more experimental confirmations, such as inspecting the phenotype of knock-out and over-expressing mutants.

## Supplementary information

Supplementary Information

Supplementary Information
