# Supplementary material for: Genome-wide analysis of fatty acid desaturase genes in rice (Oryza sativa L.)
Source: Sci Rep. 2019 Dec 19;9:19445. doi: 10.1038/s41598-019-55648-z (PMC6923433; doi:10.1038/s41598-019-55648-z)
Supplement: Supplementary file 1 — Supplementary Information [file 41598_2019_55648_MOESM1_ESM.pdf]

Article

Genome-wide analysis of fatty acid desaturase genes in rice (*Oryza sativa* L.)

Zhiguo E [1], Chen Chen [2], Jinyu Yang [1], Hanhua Tong [1], Tingting Li [1], Lei Wang [1], Hongqi Chen [1,*]

1 Key Laboratory of Rice Biology, China National Rice Research Institute, Hangzhou 310006, China; ezhiguo@caas.cn (Z.E.); 1029795028@qq.com (J.Y.); htonghz@126.com (H.T.); littgui@163.com (T.L.); wanglei05@caas.cn (L.W.)
2 Key Laboratory of Plant Functional Genomics, Ministry of Education/Key Laboratory of Crop Genetics and Physiology of Jiangsu Province/College of Agriculture, Yangzhou University, Yangzhou 225009, China; chenchen@yzu.edu.cn (C.C.)
* Correspondence: chqhzfy@126.com

Additional Information

Appendix A

S1 Fig. Phylogenetic analysis of desaturase genes in rice and *Arabidopsis*. The phylogenetic tree of all desaturase genes from *Arabidopsis* and rice after multiple sequence alignment using the full-length protein sequences is constructed by maximum likelihood method. Scale bar represents 1 amino acid substitution per site. The branches of different subfamilies are marked by different colors. The MEGA software (version 7.0.25) was used to generate the phylogenetic tree.

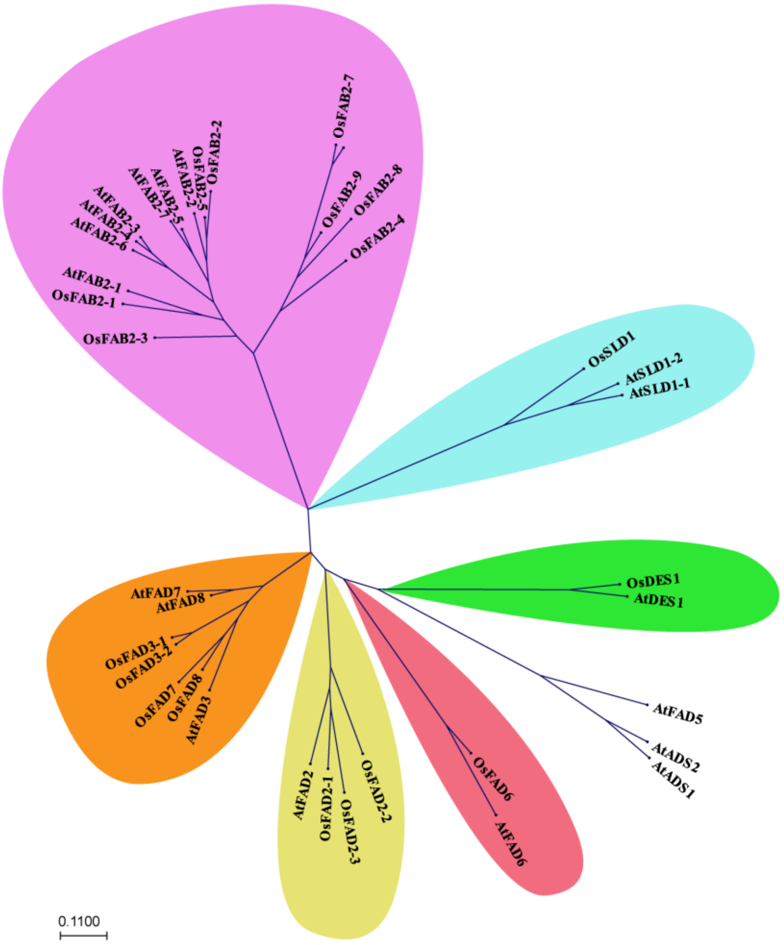

Appendix B

Table S1. Promoter analysis of nine stress or hormone-responsive desaturase genes.

Please refer to the separate CSV file (Table S1.csv).

Table S2. Primers used in qRT-PCR of desaturase genes.

Genes	Primers used in qRT-PCR(5′→3′)	
OsFAD2-1	Forwards:	GGCTGGTGCACATCTTCGTG
	Reverse:	CGCTCCCGGTCGTTGTAGAT
OsFAD2-2	Forwards:	ACAACCTCGTGGGCCTAGTG
	Reverse:	GCAGCGCGGACTTCTTCTTC
OsFAD2-3	Forwards:	GCTCCACGACCTTGCCATTG
	Reverse:	GAGGGTGTCGTCGAGGAGTG
OsFAD2-4	Forwards:	CTCGGTCTGGTCCTGCACTT
	Reverse:	GAGTTCCACGGCAGATCGGA
OsFAD3-1	Forwards:	ATTCGTGATGTGGCTTGATTTG
	Reverse:	TTGCTGCCTTTGTTGCTTCTA
OsFAD6	Forwards:	TTTTGGATGAGCACCTTTAC
	Reverse:	ATGGAATCATAGGCAGCAC
OsFAD7	Forwards:	CACAGGACGCACCACCAGAACC
	Reverse:	GGAATGCCAGCATGGGAAACG
OsFAD8	Forwards:	TGCTCGCCTACCCATTCTACCT
	Reverse:	TGTCAGCACATCGTTCCTTTCG
OsFAB2-1	Forwards:	GGCGACATCCTCGGCAAGTA
	Reverse:	CGCTCCTGGAAGCTGGTGTA
OsFAB2-2	Forwards:	GCCTGCCCACCTGATGTT
	Reverse:	TTGCGCCTTGTTTCCCTC
OsFAB2-3	Forwards:	GACCCGGACTACACGGTCAG
	Reverse:	CTCGCGACGAGGAACTCGAT
OsFAB2-4	Forwards:	GACATGCGCCAGGTCGAGG
	Reverse:	CGGACGCCATCGTGTAGCC
OsFAB2-5	Forwards:	TGACGGCAAGGACGACAACT
	Reverse:	CCCGGTGAGATCTGCGACTT
OsFAB2-6	Forwards:	AGGAGAACCACCACGACGAC
	Reverse:	TCCACCTGGCGCATGTCAA
OsFAB2-7	Forwards:	CGGTGGATTTGCAGGTGGTC
	Reverse:	CAAGGCGGCCGGAGAGATAC

OsFAB2-8	Forwards：	CTACACCCGCATCGTCTCCA
	Reverse：	GAGCAGTAGTCCGACACCGT
OsFAB2-9	Forwards：	GGGAATCCTCGAGCACCTGA
	Reverse：	TGGGCCTTCTCCTCCATCCT
OsDES1	Forwards：	GACTTCTTCTGGTCGTACACGGA
	Reverse：	GGGCCGAAGAGCTCCTTGAT
OsSLD1	Forwards：	GTTTCGCCATTTGTTCGTGACCT
	Reverse：	GCAGCAGCCCTCAATGTCTTCC
